# An Overview of the Common Elements of Learning Management System Policies in Higher Education Institutions

**DOI:** 10.1007/s11528-022-00752-7

**Published:** 2022-07-05

**Authors:** Darren Turnbull, Ritesh Chugh, Jo Luck

**Affiliations:** School of Engineering and Technology, Central Queensland University, 554-700 Yaamba Rd., Norman Gardens, Rockhampton, QLD 4701 Australia

**Keywords:** Learning management system, LMS policy, Virtual learning environment, Online learning policy, University digital policy

## Abstract

Learning management systems form an integral part of the learning environments of most universities and support a wide range of diverse activities and operations. However, learning management systems are often regulated by institutional policies that address the general use of Information Technology and Communication services rather than specific learning management system policies. Hence, we propose that learning management system environments are complex techno-social systems that require dedicated standalone policies to regulate their operation. This preliminary study examined a selection of learning management system policies from twenty universities in four countries to identify some of the elements that are considered necessary for inclusion in policy documents. Seventeen individual elements of learning management system policy documents were identified from a synthesis of the policies. These were classified into six policy categories: Accounts, Courses, Ownership, Support, Usage, and Protection. The study also identified three additional qualities of learning management system policy documents: standalone comprehensibility, platform-neutral statements, and contemporary relevance. The findings of this study will serve as a useful template for developing dedicated standalone policies for the governance of university learning management systems.

## Introduction

Learning management systems (LMS) are online software systems used to support various instructional, learning and assessment activities, and are central elements of many university course delivery systems (Turnbull et al., 2021; Weaver et al., 2008; Yueh & Hsu, 2008). The management and administration of LMSs is usually a centralized function in universities and other higher education institutions. Like other critical aspects of university operations, the effective administration of institutional LMSs depends on creating and communicating effective policies governing their use (Naveh et al., 2010). However, many universities do not have explicit policies dedicated to defining the parameters of LMS operations, such as acceptable codes of conduct for users engaging with these systems (Mohammadi et al., 2021). Instead, many institutions rely on generic policies covering Information Technology and Communication infrastructure. These policies do not necessarily cater to the LMS environment's unique characteristics as a complex, interrelated social system that technology-focused, infrastructure-heavy regulations cannot efficiently govern. Hence, there is a need for the development of dedicated LMS policies that not only address the technological environment of LMS platforms but also consider the human factors associated with people-to-people exchanges within this environment. This paper examines a cross-section of twenty LMS policies in universities from four countries with the aim of discovering some of the contemporary elements of policy development that emphasize the unique nature of LMS environments. In essence, this preliminary study is a snapshot of a cross-section of LMS policies in some of the world’s prominent higher education institutions.

The function of policy in an organization is to formally promulgate standard approaches to managing essential issues for its diverse stakeholders. A policy document is the tangible manifestation of rules and protocols that convey specific messages to various parties (von Solms & von Solms, 2004). A policy can best be understood in the context of the institutions and social relationships that give them purpose (Mosse, 2004). However, policy formation processes in organizations are often criticized because they lack consultative approaches that adequately consider the interests of the intended recipients of policy documents. Moreover, effective policies should recognize the changing and uncertain characteristics of the phenomena they address and be sufficiently malleable to accommodate substantial change. For universities that often exist and thrive in a perpetual state of flux, particular care must be taken to ensure that policy documents can survive technological and pedagogical upheavals that may impact their communities. The advent of COVID-19 has laid bare the necessity for universities to develop contingency plans to deal with major operational disrupters such as global pandemics (Rodrigues et al., 2020). LMS technology, associated pedagogical practices, and instructional design are proving to be critical elements in adapting to the post-COVID learning and teaching landscape. A key question that merits attention is whether the policies that drive the administration and operation of LMSs are up to this challenge?

An LMS learning environment is a complex techno-social system that requires institutional guidance from many diverse perspectives. At the core of an LMS’s existence is the technology that facilitates the functions necessary to carry out educational activities. These functions include learning material dissemination, stakeholder communication, student grading, progress monitoring, and records maintenance (Fathema et al., 2015; Turnbull et al., 2019). In choosing an LMS, universities can outsource their requirements to an external provider (such as Canvas) or adapt an open-source solution (such as Moodle). Open source LMSs offer the advantage of freely available source code that universities can adapt to suit their specific circumstances, and are unencumbered by recurring license fee expenses (Dobre, 2015). Conversely, proprietary solutions come bundled with ready-made quality tested modules, are relatively easy to deploy, and are supplied with ongoing technical support (Breskich et al., 2021). In either case, universities would need to develop appropriate policies to govern critical aspects of LMS use, such as mandatory adoption of an LMS platform by lecturers, the dissemination of learning materials, online announcements within the LMS, the establishment of discussion forums, the conduct of quizzes and tests, and the provision of feedback to students (Rafi et al., 2015).

University administrators must also consider whether established policies on general university issues need to be adjusted to accommodate the operation of LMSs in their unique cyber environment. For example, LMSs are capable of generating, via automated processes, vast amounts of student data that can be stored, analyzed and repurposed. However, many universities lack privacy policies that specifically address how this data will be used to inform organizational processes at a broad level (Brown & Klein, 2020). Another area of concern is intellectual property and the rights afforded to individuals and institutions that host material online. For LMSs based on open-source solutions, determining ownership of learning materials could be a simple trade-off between institutional and personal interests. However, this could be problematic for proprietary systems because the provider may have hosting terms and conditions that require materials and courses to be created to meet their corporate objectives (Pierson et al., 2013). To overcome this, universities could negotiate specific clauses in hosting agreements that clearly define ownership rights of course materials and other considerations such as acceptable use of course content by end-users engaged with the system (Pierson et al., 2013). Other general policy issues that could impact LMS use include respectful communication, sexual harassment, discrimination, and plagiarism. Governing bodies at universities have a duty of care to ensure that a regulatory framework is appropriately represented in the policy documents created for LMS use.

This paper presents the preliminary findings of a cross-section of prominent universities in four countries. The following section outlines the methodology adopted to select a cross-section of university LMS policies and analyze their content. In the results section, each selected policy's major elements are presented and coded into seventeen policy elements, further consolidated into six distinct categories. Next, the analysis and discussion section considers these policy elements in the context of LMS policy practice. This is followed by an implication for future policy section that presents a strategy for universities to create adaptive policy documents governing the adoption, maintenance, and use of university LMSs. The final section encapsulates the main arguments for creating dedicated LMS policies, while recommending that future research could help overcome the limitations of this study and contribute to the identification of other important policy elements that could be included in LMS policy documents.

## Methods

The main aim of this study is encapsulated in the following research question: What are the main elements of university policies on LMS deployment and use that regulate the management of LMSs and control user access? In answering this question, it was decided to select a sample of publicly available online LMS policy documents from universities. The approach taken was to examine five prominent universities in each of four English-speaking countries: the United States of America (USA), the United Kingdom (UK), Canada, and Australia. The 2020 university rankings from the Times Higher Education World University Rankings publication were used to locate institutions in these countries. This annual publication ranks the world’s universities based on a weighted score comprising industry income, international diversity, teaching, research, and citations (Times Higher Education World University Rankings, 2020). Apart from the time and resource constraints required to keep the review manageable, these countries were selected because of similarities in the administrative structures of their universities and the policy-making processes that support the public dissemination of university policies. Another reason for selecting these universities was that their policies were available online in English.

Working down the list of universities in each of the four countries in order of ranking, we searched for publicly available policy documents governing LMS use. If a policy document was located, it was further examined to determine if it had sufficient detail and relevance to be included in this study. To be included, the selected policy documents had to address issues relating to general LMS use or focus on particular LMS platforms such as Moodle or Canvas. Policy documents created by LMS vendors and adopted by universities without alteration were rejected, as were documents that relied on general IT usage and acceptance without specifically mentioning LMS issues. All policies had to be accessible from university public websites. The advice from the Australian Law Commission is that information available on public websites that is not encrypted is a “generally available publication” (Australian Law Reform Commission, 2010). Prior studies of university security policies have successfully used this approach (Brown & Klein, 2020; Doherty et al., 2009). After a university was rejected, the next highest-ranking university in that country was selected for examination. This process continued until policy documents from five universities in each country were identified. In total, twenty policy documents were selected for analysis after rejecting ten policies that did not meet the selection criteria. Document analysis was primarily used to examine the content of the university policies. Document analysis involves the selection and appraisal of information from documents for synthesis into discrete categories (Bowen, 2009). In the context of this study, document analysis involved the systematic analysis of web-based policy documents and subsequent synthesis of policy elements into common categories. The results of this process are outlined in the following section.

## Results

**Fig. 1:**
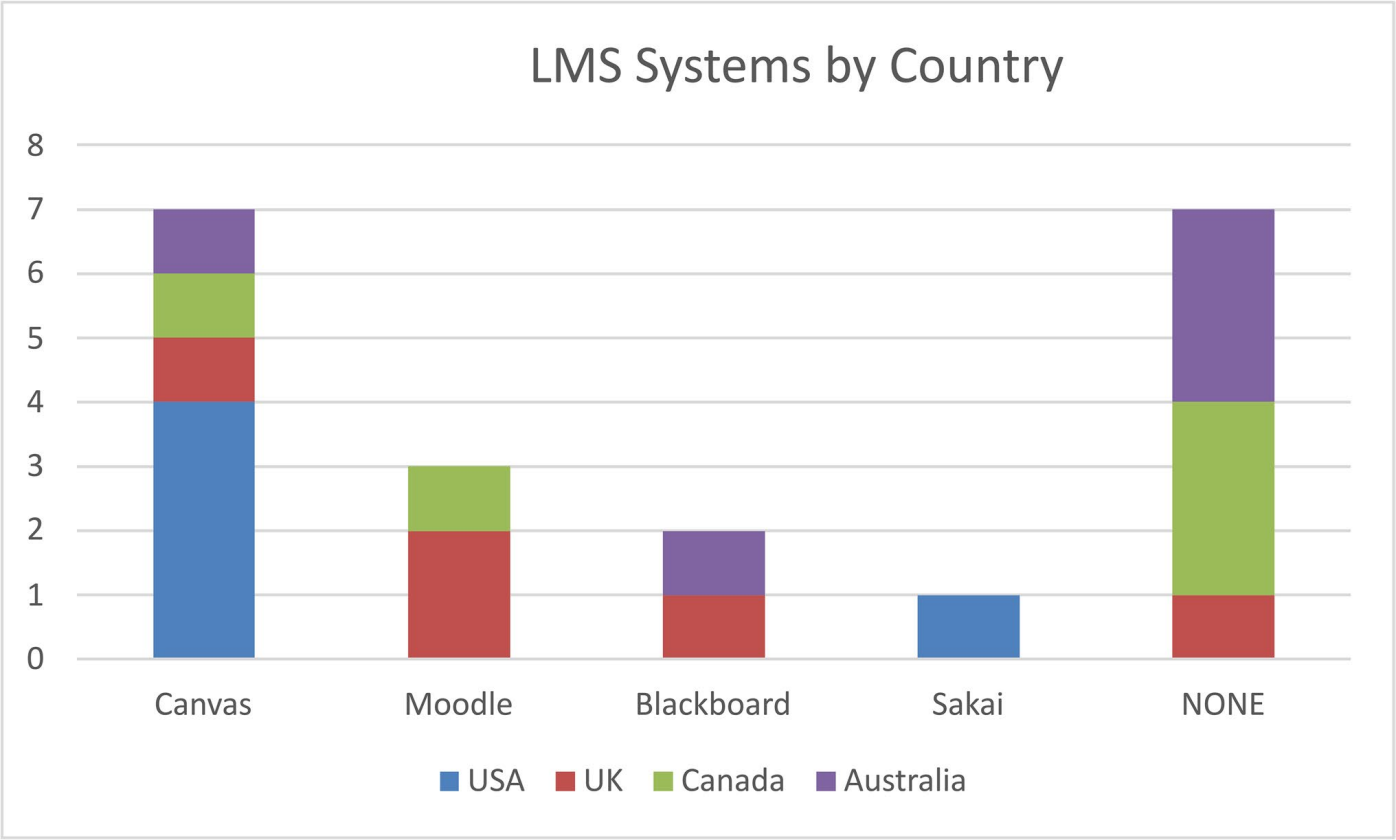
LMS distribution by country

Table 1 outlines the significant components of each university’s LMS policy. A commonly used abbreviation identifies each university. The last column of the table, LMS, indicates the name of any LMS platform referred to in the policy. If there was no mention of a particular platform, ‘None’ is indicated. A graphic representation of the distribution of LMS systems by country is displayed in Fig. 1.

**Table 1:** University LMS policies and their major elements

Ranking	Institution	Policy	Significant Policy Elements	LMS
**USA**				
2	Caltech *California Institute of Technology*	Caltech Acceptable Use Policy for the Canvas Learning Management System, operated by Instructure, Inc (California Institute of Technology, 2020)	• User license information • Ownership of contents (belongs to client) • Content Sharing • Third-party property disclaimer • Canvas copyright protection • Infringement • Service termination	Canvas
4	SUSM *Stanford University (School of Medicine)*	Course Content Access and Appropriate Use Policy (Stanford Medicine, 2021)	• Use restricted to personal academic study and review purposes • Copyright protected material • Implications for student and faculty use separately explained • Access to Canvas • Addition reference documents indicated: provost statement on copyright, honour code, fair use of materials	Canvas
7	HU *Harvard University*	Harvard Acceptable Use Policy for Canvas (Harvard University, 2020)	• Restrictions on use – only for educational and research purposes • User accounts – no use of other’s accounts or using fraudulent ID to create one • User responsible for content • Conduct – including guidance on what constitutes unacceptable posts and warnings on sharing assessment solutions with other students • Third-party sites – Harvard is not responsible for content here • Request to report all copyright infringements	Canvas
11	Penn *University of Pennsylvania*	Canvas at Penn (University of Pennsylvania, 2017)	• Acceptable use • System maintenance and upgrades • Course site enrolments • End-of-semester activities • Using Canvas for Non-SRS Sites • Learning Tools Interoperability (LTI) policy for Canvas • LMS consultations • Canvas help policy	Canvas
20	DU *Duke University*	Duke Sakai Appropriate Use Policy (Duke University, 2020)	• Terms and conditions for project sites • User roles and access • Communication and technical administration of Sakai • Conditions for creating and maintaining Sakai course sites • Copyright issues • Intellectual property • Related computing policies • Course site retention policies • Data Backup and recovery	Sakai
**UK**				
1	UO *University of Oxford*	Canvas VLE terms of use for academic and administrative staff (University of Oxford, 2019)	• Duration and access to Canvas • Acceptable use stipulations • Other uses policy such as exams • Responsibilities of local managers • Monitoring user activity • Problem identification and escalation issues	Canvas
3	UC *University of Cambridge*	Cambridge Learning Management System (CLMS) Platform Terms of Use (Cambridge University Press, 2019)	• General parameters of agreement • Intellectual property notice • Privacy notice • User accounts and responsibilities – including the need to keep private passwords and user IDs • Access by minors • E-commerce purchases via the platform • Uploading content • Message boards and forum use • Linking other sources such as home pages to the platform • Third-party links disclaimer of responsibility • Trademarks • Application of English law	None
15	UCL *University College of London*	Moodle Accessibility Statement (University College of London, 2020)	• Course structure • Orientation • Communication • Assessment • Resources • Cross-platform compatibility • Accessibility • Legal • Student active participation • Quality assurance	Moodle
27	LSE *London School of Economics and Political Science*	LSE Moodle Terms of Use (London School of Economics & Political Science, 2020)	• Moodle usage parameters • Security arrangements • Advice on responsibility for content • Data protection • Data storage • Disciplinary action	Moodle
87	UB *University of Bristol*	Blackboard Policies (University of Bristol, 2021)	• Acceptable use • Accessibility • Archive and recycle • Help response time • Copyright • Data protection • Deletion: file/course • External user accounts • Student enrolment • Service downtime • Unenrolling • Use and user details • Content System—takedown • Content system—preservation	Blackboard
**Canada**				
18	UT *University of Toronto*	Quercus and Related Technologies in the Academic Toolbox Administrative Access and Confidentiality Agreement (University of Toronto, 2018)	Policy scope constrained to administrators only: • Access rights • Password protection • Use of mobile devices to access the system • Conflicts of interests due to possible student role	None
34	UBC *University of British Columbia*	Canvas and Privacy (University of British Columbia, 2021)	• Information stored in Canvas • Mention of the law protecting privacy • Access to information in Canvas • No opt-out arrangements for students • Privacy Impact Assessment	Canvas
341-400th	UVic *University of Victoria*	LMS policy (University of Victoria, 2021)	• Access arrangements • Role of technology-integrated learning • Ownership and responsibility for course content	None
601-800th	Concordia *Concordia University*	Security Guidelines (Concordia University, 2021)	• Access to records in Moodle by employees • Password requirements • Moodle should not be left logged on unattended • Printed documents from Moodle must be kept in a safe place • Misuse of Moodle must be reported to the Office of the Provost	Moodle
601-800th	UR *University of Regina*	UR Course Terms of Use (University of Regina, 2021)	• Guidance to instructors to ensure materials posted to system • Guidelines for copyright and fair dealings • Guidance to students on the use of material for personal use (not commercial)	None
**Australia**				
66	UQ *University of Queensland*	eLearning procedure (The University of Queensland, 2021)	• Si-Net courses • LMS site components • Archiving arrangements • Material availability • Roles and responsibilities of users • Monitoring arrangements • Recording and reporting • Guest access • Minimum presence	None
75	Monash *Monash University*	Service Agreement (Monash University Studies Online, 2019)	• Service provider responsibilities • Client responsibilities • Infrastructure and support	Blackboard
120	UA *University of Adelaide*	Schedule B: Minimum use of MyUni (The University of Adelaide, 2018)	• Expected elements of MyUni courses • Must be published one week prior to commencement of course start date • Communications to be maintained within LMS environment • Copyright issues • Compliance exemptions	None
193	UCan^1^ *University of Canberra*	UCLearn (Canvas) teaching site publishing procedures (University of Canberra, 2019)	• Access rights and elevating user roles • Teaching site creation • Retention of records • Publishing material to the site • Roles and responsibilities of users	Canvas
201-250th	MU *Macquarie University*	Learning Technologies Policy (Macquarie University, 2021)	• Alignment of system use (iLearn) with teaching goals of the university • Management of iLearn • Quality assurance issues • External Technologies • Compliance and breaches	None

^1^This abbreviation was modified from UC which denotes the University of Cambridge in this table.

Canvas, an LMS designed and maintained by Instructure, was the most mentioned LMS platform in the examined policies. Instructure is a USA-based company founded in 2008 whose principal business model is vested in providing LMS solutions via Canvas (Instructure, 2021). Instructure maintains Canvas systems in all four countries to a varying degree but is most prominently represented in the policies of the US institutions: Caltech, HU and SUSM. The next most featured LMS was Moodle, an open-source system adopted by the LSE and UCL in the UK, and in Canada by Concordia. Blackboard, a proprietary system, was referenced in the policy documents in Monash in Australia and UB in the UK. Duke University’s Sakai-based policy was the only open-source system referenced in the USA documents.

**Fig. 2:**
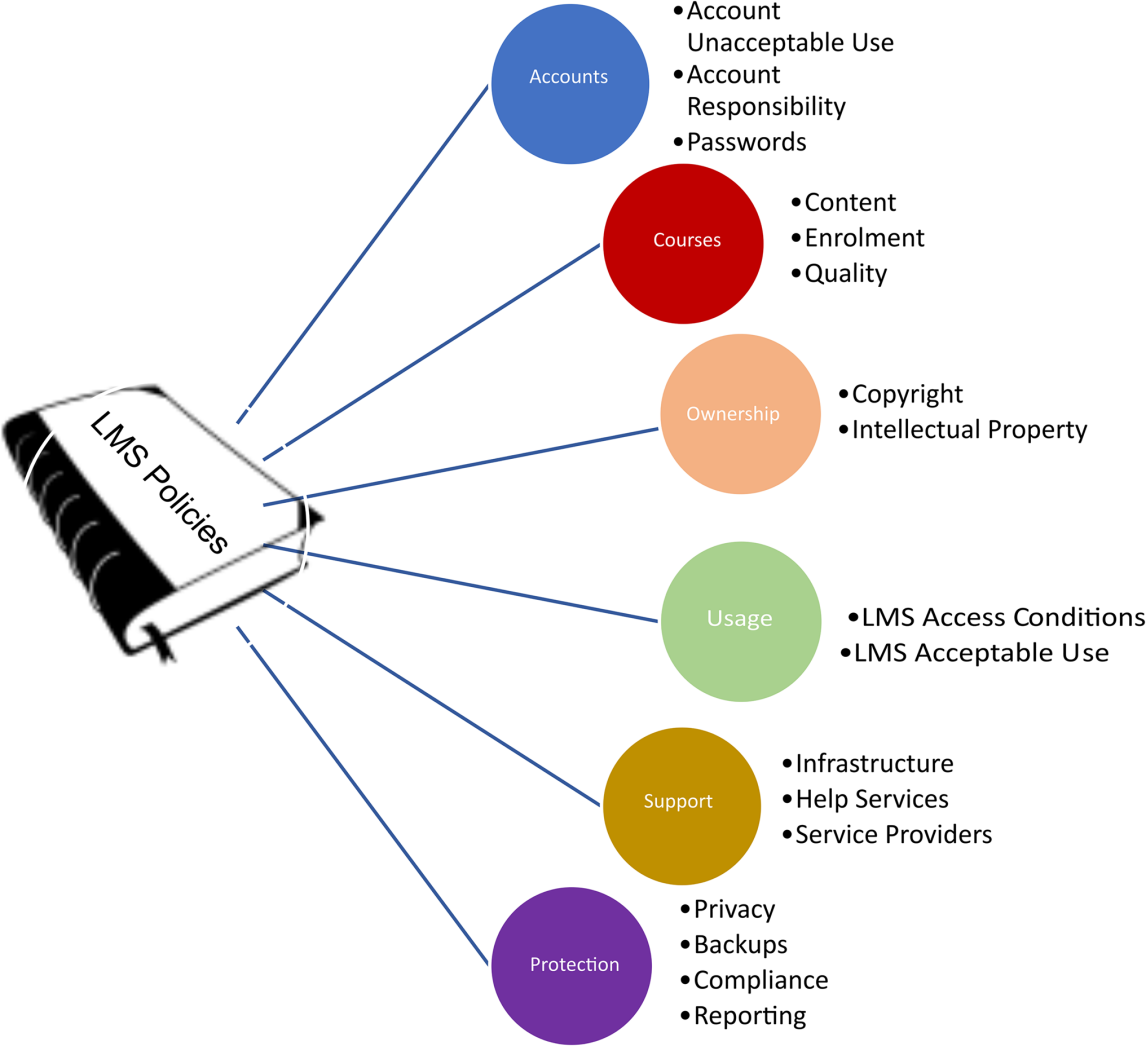
LMS policy elements by category

The major policy elements identified in Table 1 were further examined in each document to identify a set of elements that could describe the entire range of issues collectively presented by the entire document sample. Using an iterative process, *seventeen elements* were eventually identified, and these were further segmented into *six broad policy categories,* as shown in Fig. 2.

The relative merits of these elements in contributing to effective LMS policy formation form the basis of the discussion presented in the following section.

## Analysis and Discussion

**Fig. 3:**
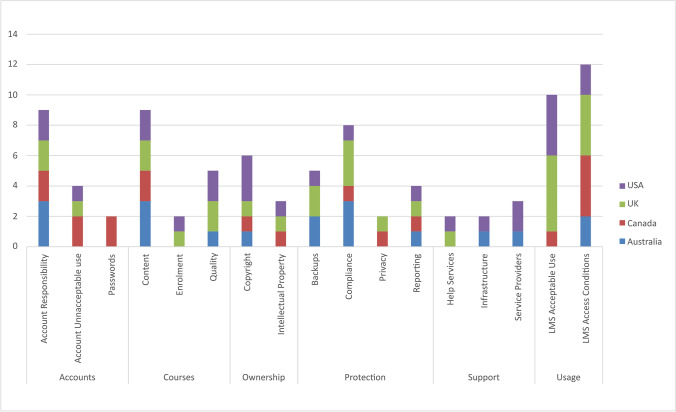
Distribution of policy elements by country of origin

Figure 3 displays a distribution of derived policy elements substantially identified in each university’s LMS-related policy publication.

These elements and their relevance to LMS policy formation are presented in the context of the six policy categories in the following discussion. Institutions mentioned in the discussion are also referred to by their commonly used abbreviation or short form, as defined in Table 1.

### Accounts (Account Responsibility, Account Unacceptable Use, and Passwords)

The primary way that access is granted to users of online resources is to authenticate the unique user accounts and passwords assigned to each user. This name-password combination represents the identity of the individual to be granted access to available resources (Shim et al., 2005). For LMSs, the user account also defines the role or function that the user can perform within the system. For example, there are different classes of users, such as students, administrators and teachers—all of whom are granted different access rights based on an assessment of their needs (Luminita, 2011). Both authentication (establishing the identity of a user) and authorization (granting permission to access specific resources or functions) are key security features that regulate LMS operations. Policies from both the University of Toronto and Concordia University had explicit statements that dealt with the formation and/or maintenance of user account passwords for their LMSs. It is certainly possible that the other institutions had policies that dealt with passwords that were embedded in more general policies. This is because many university information systems are integrated with a single sign-on, so separate LMS policies on user account characteristics are not necessary.

Responsibility for account use was a rather broad area that was addressed in the policies of nine institutions from all four countries: USA (Caltech, HU), UK (UO, UC), Canada (UBC, UT), and Australia (UQ, Monash, UCan). Issues covered here included resource sharing conditions such as restrictions on certain content, including viruses, defamatory materials and documents that might breach copyright regulations, content ownership and implied permissions granted to hosting services once materials are uploaded, specific account owner responsibilities by role, and access conditions such as obtaining parental consent if the user is not an adult.

In the final element in this category, unacceptable use, four universities provided clear examples of inappropriate usage (HU, UT, Concordia, UT). These included the use of fraudulent identification to create user accounts, allowing other people to access user passwords, potential conflicts of interest arising from student users granted administrator rights, and leaving logged-on accounts unattended.

### Courses (Content, Enrollment, Quality)

The production, deployment, and maintenance of course-related assets within an LMS is a complex activity and difficult to codify in a policy document in a manner that applies to all users and situations. Nevertheless, numerous examples of course-related policy statements were cited in the reviewed documents. Guidelines on course content requirements, how to upload materials, content ownership, and student use of course content were featured elements of nine policies in all four countries: USA (Caltech, Duke), UK (UC, UCL), Canada (UR, UVic), and Australia (UQ, UA, UCan). Enrollment guidelines were mentioned in two policy documents: the University of Bristol specified that most students were enrolled automatically in the LMS based on registration in other student information systems, and the University of Pennsylvania provided details of procedures for enrolling students, faculty, and teaching assistants as separate groups of users. The final element in this category, course quality, was covered by several policies and included end-of-course procedures and general course quality issues such as removing old course content (University of Bristol), preserving a copy of gradebook information at the end of the semester (Duke University), the provision of resources to maintain course development quality (Macquarie University), advice on navigation aids to include in course materials (University College of London), and advice on acceptable reasons for concluding courses (University of Pennsylvania). End-of-course procedures are included in the quality category because of their importance in preserving the integrity of the information gathered over the duration of a course.

### Ownership (Copyright, Intellectual Property)

The terms ‘intellectual property’ and ‘copyright’ are related concepts. Copyright in a university context refers to a legal right to distribute, reproduce, or sell academic works such as books, syllabi, and scholarly publications. By contrast, intellectual property encompasses principles of copyright but is considered a more comprehensive term that is particularly suited to dealing with issues raised by online ownership (Masson, 2010). In a sense, copyright issues could be considered subordinate to the principle of intellectual property. Policies from three universities made explicit reference to intellectual property or materials ownership (UC, Duke, UVic). Copyright was included as a major section in the LMS policy documents of six universities (Caltech, HU, SUSM, UB, UR, UA). The choice of the label ‘copyright’ instead of ‘intellectual property’ to convey ownership information is interesting. Nadel (2004) contends in his article investigating the impact of copyright law on creative output that the existing legal framework governing copyright may be responsible for reducing the incentive to create and share intellectual property. The term ‘copyright’ tends to be associated with the legal consequences of non-compliance, whereas ‘intellectual property’ is generally interpreted as a non-threatening descriptor of *creations of the mind*. Avoiding the use of language with punitive overtones such as *copyright restrictions* to identify the ownership of intellectual property may assist in promoting an environment that encourages the open exchange of ideas within the structured environment of modern LMSs.

### Usage (LMS Acceptable Use, LMS Access Conditions)

LMS usage issues are a core concern of universities and represent the largest category of elements identified in the policy documents. Conditions that define acceptable parameters of LMS use, such as restrictions on use, communication regulations, conditions of sale for e-commerce purchases made via institutional LMSs, and fair use provisions, featured prominently in ten policies from institutions in three countries: USA (Caltech, HU, SUSM, DU), UK (UO, UC, UCL, LSE, UB) and Canada (UR). Access conditions that define the circumstances under which users may attempt to locate resources within the LMS environment are closely related to acceptable use. Examples of such conditions featured in the policies include user rights of access, access rights according to user role, and access duration limitations. These were prevalent in the policies in 11 institutions from all four countries: USA (HU, SUSM, Duke), UK (UO, UC, UB), Canada (UT, UBC, Concordia) and Australia (UC, UCan).

### Support (Infrastructure, Help Services, Service Providers)

Support in this context refers to the resources and effort required to maintain a satisfactory level of LMS service to university communities. This could be provided externally by the LMS vendor or the university itself. LMSs are complex learning environments that require ongoing support to ensure their continued effectiveness. For end-users in particular, one of the essential ingredients to maintaining a positive engagement with LMS services is the knowledge and expectation that technical support is available when needed (Alshammari et al., 2016). The examined papers demonstrated support for LMS operations in three areas: infrastructure, help services, and service providers. Infrastructure support was an element applicable to the policies of two universities and included guidance on system maintenance and upgrade processes and other support issues (Penn, Monash). The University of Pennsylvania’s Canvas usage policy outlined conditions for providing help services to users of their Canvas system, while the University of Bristol provided a response-time policy for help requests from users of their LMS. Three universities (Caltech, DU, Monash) included information on service provider responsibilities in their policies. The support required to service LMS operations that ensure the user community is fully engaged with online learning environments is complex and subject to the unique operating conditions of each university. The cross-section of support-related statements identified in the policy documents indicates that institutional support is essential regardless of whether the adopted LMS platform is an open-source solution maintained by the university or an external proprietary system.

### Protection (Privacy, Backups, Compliance, Reporting)

The protection category of elements comprises policy statements on privacy, information backups, compliance initiatives, and reporting mechanisms for instances of policy breaches. Data protection in the form of user data privacy is essential information that should be included in LMS policy statements. Universities have a duty of care to ensure that student interests are central to the formation of data privacy policies and practices that ensure LMS users are able to control how they engage with the technology, are non-discriminatory, and make data extraction and use transparent to the user community (Brown & Klein, 2020). One UK university, UC, and one Canadian university, UBC, explicitly dealt with privacy issues in their policies. Backups, the second element in this category, included statements from the policies of five universities on a variety of dimensions, including backup and recovery, course archiving, student record retention, and data storage (DU, LSE, UQ, UB, UCan). The compliance element that deals with breaches of acceptable usage rules was a prominent feature of eight university policies (Caltech, UO, UC, LSE, Concordia, UQ, UA, Macquarie). Policy statements in these documents included compliance guidelines, disciplinary actions, relevant applicable laws, and compliance exemptions. Reporting mechanisms for unauthorized usage of LMSs were covered in four policy documents (HU, UO, Concordia, UQ). Issues raised in these documents included reporting protocols to be followed in response to breaches of acceptable content rules, the need to report instances of copyright infringements when discovered, misuse of information by system users, and the circumstances under which reports on policy non-compliance are systematically provided to responsible committees. In summary, it was apparent that protection issues were a prominent concern of policymakers at the universities investigated, with multiple distinct inclusions of protection-related policy elements in universities from the four countries considered in this study.

## Implications for Future Policy

This study has sought to explore self-contained university policy documents dedicated to LMS use that contain standalone statements regulating LMS administration and user engagement. In the course of our analysis of the 20 selected universities in Table 1, it was discovered that some universities simply published material (or links to materials) that point to vendor documentation relevant to their product, such as Instructure’s Connect. All of the policy statements on LMSs listed in Table 1 are directed towards LMS administration and use and reinforce our argument that complex techno-social systems like LMSs require policy frameworks in their own right. By identifying common threads throughout all of the documents considered in this study, a list of possible components for an LMS policy template was developed, as indicated in Fig. 2. This study has also highlighted additional non-content-related qualities of good LMS policy design worthy of inclusion in policy-making procedures. First, policies should be self-contained with sufficient detail in plain English to convey key information, so that reference to other documents is not necessary to understand particular policy elements. For example, the University of Regina’s web-based policy statement relating to the use of copyright material only contains a link to a general university policy on copyright (University of Regina, 2021) rather than explaining what copyright means for users of an LMS. By contrast, Caltech’s LMS copyright statements clearly define how these apply to the Canvas LMS in an organizational context (California Institute of Technology, 2020). Second, it is preferable to ensure that policy statements are platform-neutral. In other words, they do not embed the names or titles of specific LMSs within the document text. The University of Bristol’s LMS policy, while including many of the elements identified in this study, is structured around the Blackboard proprietary system and refers to ‘Blackboard’ as a synonym for all LMSs. This may serve to entrench Blackboard as the only term relevant to LMS deployment for stakeholders at the university, limiting or voiding the applicability of the Blackboard-based policy to other platforms that may be adopted in the future. A more technology-agnostic approach, such as Cambridge’s CLMS policy, maintains the focus of policy elements on the institution and its functions rather than the tool and the vendor. Finally, policy documents should be constructed in such a way that they can be easily modified and adapted to abrupt changes in circumstances that are difficult to anticipate (Walker et al., 2001). For example, the COVID-19 pandemic has forced many educational institutions to adopt digital learning as a necessary emergency measure to compensate for the lack of face-to-face instruction which has, in turn, led to the rapid development of policies and functional plans that could address the potential victimization of disadvantaged students coping with the new requirements of online learning (Karakose, 2021). Given the ongoing impact of COVID-19 on university operations worldwide, a thorough revision or replacement of LMS policies (should they exist) could improve their relevance to current operations.

## Conclusion

Contemporary LMSs are complex techno-social systems that support a broad range of educational activities in modern universities. Policy documents are the principal means for university administrations to convey important information to stakeholders on how the adoption, maintenance, and use of these systems is to be regulated. Unfortunately, many universities neglect to develop standalone policy documents that specifically regulate the administration and use of LMSs. Rather, they rely on more generic policies such as Information Technology and Communication usage agreements, privacy regulations, and intellectual property rights to address specific issues arising from the use of complex LMSs. To help gain an understanding of the essential elements to include in an LMS policy, comparative exploration of the contents of existing LMS policy documents from a cross-section of prominent universities is a good starting point.

This preliminary study examined a snapshot of written LMS policies from twenty universities in four countries and identified seventeen elements that could be included in a policy design template. These elements were classified into six policy categories: Accounts, Courses, Ownership, Usage, Support, and Protection. In addition, three other qualities of LMS policy statements were also established: standalone comprehensibility, platform-neutral statements, and contemporary relevance. Together these categories and qualities provide a practical starting point for universities to design or enhance their LMS policy statements to better align LMS administration and use with stakeholder interests and broader community responsibilities.

This study is limited by the inclusion of English-only institutions from four countries based on a single ranking scheme. The use of additional rating metrics and expansion of the selection criteria to include prominent non-English language documents would significantly enhance the generalizability of the study. The authors also acknowledge that institutions not featuring prominently in university rankings may provide insights into LMS policy formation that could contribute to this study. Future studies could, for example, expand on our findings in this paper by exploring LMS policy formation in a cross-section of universities that specialize in providing educational services to disadvantaged communities or remote regional areas. The inclusion of internal documents, if available, could also provide valuable insights into LMS policy issues. Despite these limitations, significant insights into existing LMS policy elements were gained from this study. As Walker et al. (2001) contend, we can learn from the policy choices of others:

“Policy analysis should take into account the fact that the effects of policy choices depend on information about events that have happened and events that are yet to happen, including choices made by others.” (p. 283).

We hope this paper inspires future research into LMS policy formation to further help educational institutions design more cohesive LMS policies that address the concerns and interests of LMS stakeholders.

## Declarations

### Author Agreement

All authors have seen and approved this manuscript for submission to TechTrends.
